# Research on incentive mechanisms for veterans’ participation in grassroots governance: An analysis based on behavioral expectations

**DOI:** 10.1371/journal.pone.0351162

**Published:** 2026-07-17

**Authors:** Shuqiong Wang, Zhao Zhang

**Affiliations:** 1 School of Sociology, Guizhou Minzu University, Guiyang, Guizhou, People’s Republic of China; 2 School of Mathematical Sciences, Xingyi Normal University for Nationalities, Xingyi, Guizhou, People’s Republic of China; Chinese Culture University, TAIWAN

## Abstract

Under the modernization of grassroots governance, veterans constitute a high-quality human resource for enhancing grassroots social governance capacity. Drawing on questionnaire survey data from 525 veterans in Guizhou Province, China, this study identifies three distinct veteran groups using latent class analysis (LCA): Utilitarian (51.3%), Mission-Oriented (23.8%), and Development-Focused (24.9%). Empirical results reveal clear heterogeneity in policy responsiveness and behavioral mechanisms across groups. First, the Utilitarian group responds most strongly to Subsidies, with participation behavior predominantly driven by economic rationality. Second, the Mission-Oriented group demonstrates honor-driven motivations, with symbolic recognition playing a central role in stimulating governance engagement. Third, the Development-Focused group prioritizes career advancement and exhibits significant responsiveness to promotion-related incentives. Based on these findings, this study constructs a mediation model of “Policy Instruments (PI)—Behavioral Expectations (BE)—Participation Behavior (PB)” and proposes an intervention pathway centered on “expectation diagnosis” and “instrument matching.” By revealing the internal mechanism through which policy instruments (PI) influence participation behavior (PB) via Behavioral expectations (BE), this research provides empirical evidence and theoretical insights for shifting incentive strategies from “uniform provision” to “precision adaptation.”.

## 1. Introduction

Grassroots governance serves as the cornerstone of modern governance systems and plays a decisive role in translating public policies into tangible social outcomes. In many countries, increasingly complex social structures, demographic shifts, and governance fragmentation have intensified the demand for diverse governance actors beyond traditional administrative frameworks [1–3]. In this context, veterans—with their organizational discipline, crisis response capabilities, and collective orientation cultivated during military service—have emerged as a distinctive group capable of injecting vitality into grassroots governance, garnering growing attention from both academia and policymakers [4–6].

International research on veterans’ post-service participation has primarily focused on three interrelated themes. First, studies on veteran reintegration highlight the complex psychological and social challenges associated with the transition from military to civilian life [7–9]. Recent research emphasizes that successful reintegration requires not only institutional support but also strong community engagement and identity reconstruction [10–12]. These studies demonstrate that veterans’ post-service roles are shaped by both social networks and institutional opportunities. Second, a growing body of literature examines civic and governance participation. Research on public participation indicates that institutional arrangements and policy feedback mechanisms can significantly influence citizens’ engagement in governance activities [2,13,14], while broader political and cultural contexts may also shape patterns of civic participation [15].Within veteran populations, military socialization and shared service experiences may enhance collective identity and civic commitment, thereby facilitating participation in community governance [4,6]. Third, policy design research increasingly highlights the importance of policy instruments in shaping behavioral responses. Scholars emphasize that different types of instruments—such as economic incentives, symbolic recognition, and capacity-building measures—operate through distinct behavioral mechanisms [16–18]. However, the effectiveness of these instruments depends largely on how target groups perceive and interpret policy signals [19,20].

Within the Chinese context, research on veterans’ participation in grassroots governance has developed rapidly in recent years, driven by the institutionalization of veterans’ affairs and the enactment of the Veterans Protection Law. Existing studies have examined veterans’ engagement from perspectives such as policy support, organizational embeddedness, and social role transformation [21]. In particular, research on Policy Instruments (PI) has drawn on public policy design theory to categorize incentive tools into material, symbolic, and developmental types [16–17]. Meanwhile, psychological studies have underscored the lasting influence of military identity, honor, and service ethos on veterans’ post-service behavior [22–23]. Despite these advances, two significant research gaps remain inadequately addressed.

The first gap concerns the heterogeneity within the veteran population. Most existing studies implicitly treat veterans as a relatively homogeneous group, overlooking substantial variations in their post-service expectations, career trajectories, and governance motivations. Although recent research has begun to apply Latent Class Analysis (LCA) to identify subgroups of veterans with different risk profiles or behavioral tendencies [24], such approaches have not yet been systematically integrated into governance participation research, particularly in relation to incentive design.

The second gap lies in the mismatch between Policy Instruments (PI) and Behavioral Expectations (BE). While various policy tools aim to encourage veterans’ participation in grassroots governance, their effectiveness is often hindered by a neglect of veterans’ subjective expectations. Existing studies typically assess either Policy Instruments (PI) or Behavioral Expectations (BE) in isolation, failing to elucidate how specific Policy Instruments (PI) activate—or fail to activate—the psychological mechanisms that translate into Participation Behavior (PB).

Addressing these gaps requires moving beyond purely structural or institutional explanations of incentives toward a mechanism-oriented analytical framework. This study conceptualizes veterans’ participation as a process mediated by Behavioral Expectations (BE)—that is, individuals’ subjective anticipations regarding material returns, social recognition, and personal development.

To clarify the theoretical positioning of Behavioral Expectations (BE), this study distinguishes the concept from several related constructs in behavioral research. Behavioral expectations refer to individuals’ anticipatory evaluations of the outcomes associated with a potential action. In contrast to behavioral intention in the Theory of Planned Behavior [25], which reflects an individual’s readiness to perform a behavior, BE focus on how individuals assess the potential returns of action prior to forming such intentions. BE also differ from motivation and attitudes: motivation refers to the internal forces that drive behavior, while attitudes represent relatively stable evaluative orientations. By comparison, behavioral expectations are situational and policy-sensitive assessments regarding expected outcomes. This understanding aligns with expectancy theory [26], which emphasizes that expectations about outcomes shape behavioral choices. Conceptualizing BE in this way provides a psychological mechanism linking policy instruments to participation behavior.

Unlike approaches that assume a universal model of economic rationality, this method recognizes that incentive effects depend on how different groups interpret policy signals.

It should be explicitly noted that this study does not claim causal inference in a strict experimental sense. Based on cross-sectional survey data, the proposed analytical framework focuses on identifying empirically consistent mediation pathways between Policy Instruments (PI), Behavioral Expectations (BE), and Participation Behavior (PB). Although alternative causal directions remain theoretically plausible, examining these mediation patterns provides valuable insights into how different veteran groups psychologically process Policy Instruments (PI), thereby offering an evidence-based foundation for more precise policy design.

Using questionnaire data from 525 veterans in Guizhou Province, this study integrates Latent Class Analysis (LCA) and mediation modeling to achieve three research objectives: first, to identify distinct subgroups of veterans based on their Behavioral Expectations (BE); second, to analyze the sensitivity of different groups to specific Policy Instruments (PI) and their combinations (Combined-PI); and third, to test whether Behavioral Expectations (BE) function as a mediating mechanism linking Policy Instruments (PI) to Participation Behavior (PB). Through this research approach, the study contributes to public governance research in two ways: empirically, by revealing the structural heterogeneity in veterans’ incentive responses; and theoretically, by refining—rather than replacing—existing explanations of economic rationality through an expectation-mediated perspective applicable to governance participation.

For clarity and conciseness, the following abbreviations are used throughout the remainder of this manuscript: Policy Instruments (PI), Behavioral Expectations (BE), and Participation Behavior (PB). Unless otherwise specified, these abbreviations are used consistently in all subsequent sections.

## 2. Methods

### 2.1. Definition of core constructs

The study examines three central concepts:

**Veterans** refer to military personnel who have completed active service, including discharged soldiers, non-commissioned officers, and commissioned officers. This group shares distinctive attributes: rigorous military training that develops strong discipline and teamwork abilities, specialized skills in emergency response and resource coordination, and the challenging transition from military to civilian roles requiring social readjustment.

**Grassroots Governance** encompasses public affairs management at basic administrative levels like townships, sub-districts, villages and communities. Its key features include multiple participating stakeholders (government bodies, social organizations, residents), direct handling of livelihood-related matters such as public safety and environmental maintenance, and governance approaches emphasizing consultation and co-creation.

**Incentive mechanisms** denote institutional designs aimed at motivating participation, consisting of three key dimensions: material incentives (compensation packages, welfare benefits), recognition-based incentives (honors, social acknowledgment), and Development-focused incentives (career advancement opportunities, skills training programs).

### 2.2. Survey instrument and rationale for question design

The questionnaire employed multiple measurement formats tailored to the characteristics of each construct. Behavioral Expectations (BE) were measured using a seven-point Likert scale ranging from 1 (strongly disagree) to 7 (strongly agree), capturing veterans’ expectations regarding material returns, social recognition, and personal development through items B1–B3. Policy Instrument (PI) perception was assessed using two complementary approaches: exposure frequency (never, occasional, frequent) and attractiveness evaluation on a 0–10 rating scale for specific instruments such as subsidies, honors, training, and promotion. Participation Behavior (PB) was measured using two indicators reflecting behavioral engagement: participation frequency in governance activities and the degree of decision-making involvement. This multi-format measurement design enabled the study to capture both subjective expectations and observed participation behaviors, providing an empirical basis for subsequent latent class analysis (LCA) and mediation modeling.

The questionnaire is structured as follows in Table 1

**Table 1:** Veterans’ participation in grassroots governance survey.

Instructions: This survey is solely for academic purposes and is anonymous. Completion time: ~ 12 minutes. Thank you for your support!			
**A. Demographic Information**			
**A1**. Years since discharge:			
□ < 2 □ 2-5 □ 5-10 □ > 10			
Rationale: A1(Critical timepoints affect social readjustment [19]).			
**A2**. Highest military rank:			
□ Enlisted soldier(**soldier**) □ Non-commissioned officer (**NCO**) □ Commissioned officer(**officer**)			
Rationale: A2(Rank correlates with resource acquisition capacity [27]).			
**A3**. Monthly income (CNY):			
□ < 3000 □ 3000-5000 □ 5000-8000 □ > 8000			
Rationale: A3(Controls economic status’ effect on PI sensitivity (ref. EVS scale)).			
**B. Behavioral Expectations (BE)** (7-point Likert scale: 1 = Strongly disagree, 7 = Strongly agree)			
**B1**. I hope that participation in grassroots governance can provide substantial income.			
□1 □2 □3 □4 □5 □6 □7			
Rationale: B1(Measures instrumental BE [22]).			
**B2**.I hope to improve the community’s respect for me through participation.			
□1 □2 □3 □4 □5 □6 □7			
Rationale: B2(Assesses military identity [22]).			
**B3**. I hope to improve management skills through participation			
□1 □2 □3 □4 □5 □6 □7			
Rationale: B3(Evaluates developmental BE [22]).			
**C. Policy Instruments (PI)**			
**C1**. Exposure frequency (past 2 years)			
PI	Never	Occasional	Frequent
C11 Subsidies	□	□	□
C12 Honors	□	□	□
C13 Training	□	□	□
Rationale: C1(Measures PI exposure intensity [22]).			
**C2**. Single PI attractiveness (0–10 scale)			
PI		Rating	
C21 Subsidies		□ 1 □ 2 □ 3 □ 4 □ 5 □ 6 □ 7 □ 8 □ 9 □ 10	
C22 Honors		□ 1 □ 2 □ 3 □ 4 □ 5 □ 6 □ 7 □ 8 □ 9 □ 10	
C23 Training		□ 1 □ 2 □ 3 □ 4 □ 5 □ 6 □ 7 □ 8 □ 9 □ 10	
C24 Promotion		□ 1 □ 2 □ 3 □ 4 □ 5 □ 6 □ 7 □ 8 □ 9 □ 10	
Rationale: C21(Baseline economic incentive (Art.25, China’s Veterans Support Law));			
C22(Military-specific symbolic needs [16]);C23(CLong-term human capital [19]);			
C24(Career development focus (ref. EVS)).			
**C3**. Combined-PI attractiveness (0–10 scale)			
Combined-PI		Rating	
C31 Subsidies+Honors		□ 1 □ 2 □ 3 □ 4 □ 5 □ 6 □ 7 □ 8 □ 9 □ 10	
C32 Training+Promotion		□ 1 □ 2 □ 3 □ 4 □ 5 □ 6 □ 7 □ 8 □ 9 □ 10	
C33 Subsidies+Training+Honors		□ 1 □ 2 □ 3 □ 4 □ 5 □ 6 □ 7 □ 8 □ 9 □ 10	
Rationale: C31(Material-symbolic synergy [20]);C32(Dual capability-position path [23]);			
C33(Comprehensive incentive mix [16]).			
**D. Participation Behavior (PB)**			
**D1**.Governance participation frequency (past 6 months):			
□0 □1-3 □4-6 □ > 6			
**D2**.Decision-making involvement in participated activities:			
□ < 20% □20-50% □ > 50%			
Rationale: D2(Differentiates executive vs. decision-making PB [5]).			

To ensure the credibility of subsequent analyses, reliability tests were conducted for the core modules of the survey instrument, including Behavioral Expectations (BE), Policy Instrument perception (PI), and Participation Behavior (PB). Different reliability assessment methods were applied according to the measurement structure of each construct. Cronbach’s α was used to assess the internal consistency of the reflective Behavioral Expectations (BE) scale, while Composite Reliability (CR) was employed for Policy Instruments (PI), which represent a formative construct composed of different policy tools. For Participation Behavior (PB), which contains only two indicators, the Spearman–Brown coefficient was used to evaluate reliability. The results demonstrate acceptable reliability levels for all constructs and are presented in Table 2.

**Table 2:** Reliability and validity analysis of the survey instrument.

Module	Measurement Items	Test Method	Result	Interpretation
BE	B1-B2-B3	Cronbach’s α	**α=0.76**	Acceptable internal consistency
PI	C21-C22-C23-C24	Composite Reliability (CR)	**CR = 0.71**	Meets minimum reliability standard for formative constructs
PB	D1-D2	Spearman-Brown Coefficient	**SB = 0.66**	Moderate reliability

### 2.3. Data compilation

The survey was administered via a mobile application through veterans affairs bureaus across 10 counties in Guizhou Province, yielding 525 valid responses. The frequency distributions are presented in Table 3.

**Table 3:** Survey data summary.

**Demographic Information**	A1		<2 years			2-5years			5-10 years			>10 years	
			38			142			224			121	
	A2		soldier				NCO				Officer		
			386				104				35		
	A3 (CNY)		<3000			3000-5000			5000-8000			>8000	
			255			167			75			28	
**BE**	Item		1	2		3	4		5	6		7	Mean
	B1		9	10		36	52		78	126		214	5.69
	B2		21	46		56	100		109	104		89	4.71
	B3		8	40		133	127		75	90		52	4.33
**PI**	C1		Never				Occasional				Frequent		
		C11	16				446				63		
		C12	6				421				98		
		C13	102				377				46		
	C2		1	2	3	4	5	6	7	8	9	10	Mean
		C21	1	6	8	7	20	32	79	80	157	135	8.22
		C22	22	12	20	56	61	46	73	78	56	101	6.78
		C23	33	28	20	103	125	67	55	41	11	32	5.18
		C24	8	11	20	16	22	6	11	105	115	211	8.31
	C3		1	2	3	4	5	6	7	8	9	10	Mean
		C31	0	8	4	11	5	17	20	55	106	299	9.00
		C32	1	7	3	11	38	55	53	108	124	125	7.98
		C33	0	0	0	2	9	13	13	26	100	362	9.10
**PB**	D1		0			1-3			4-6			>6	
			19			271			147			88	
	D2		<20%			20-50%			>50%				
			351			132			42				

### 2.4. Data collection protocol

The participant recruitment for this study was completed between February 15 and May 20, 2024. Throughout the research process, all data underwent rigorous anonymization procedures to ensure that researchers could not access any information that could identify individual participants. The processed dataset has been stripped of all sensitive personal information, fundamentally safeguarding participant privacy.

Prior to the study implementation, standardized ethical procedures were followed to obtain verbal informed consent. All participants provided oral confirmation of their agreement to participate under conditions of full informed consent.

### 2.5. Identification of behavioral expectation types

The veteran population exhibited significant heterogeneity. Using Latent Class Analysis (LCA) on behavioral expectation measures (B1-B2-B3) from the survey, we classified veterans into distinct subgroups. The analysis was conducted in R, with results shown in Table 4.

**Table 4:** LCA classification results.

Number of Classes	AIC	BIC	LMR p-value	Entropy
2	2002.3	2046.7	0.001	0.71
**3**	**1987.6**	**2068.2**	**0.003**	**0.84**
4	1975.4	2080.1	0.112	0.83

The selection of latent classes followed standard LCA procedures. With a significant LMR test (p < 0.05), the three-class model showed the lowest AIC and BIC values while maintaining an entropy above 0.8, indicating accurate classification.

Based on posterior probabilities obtained from LCA analysis, the 525 samples were classified into their most probable categories. The characteristics of these three categories are presented in Table 5.

**Table 5:** Characteristics of three behavioral expectation groups.

Type	Proportion	B1	B2	B3	Demographic Profile	
Utilitarian	51.3%	High **(6.2)**	Low (3.8)	Medium (3.9)	87%	soldiers
					68%	discharged <5 years
Mission-Oriented	23.8%	Medium (4.5)	High **(5.5)**	Low (4.4)	59%	officers
					72%	discharged >10 years
Development- Focused	24.9%	Low (4.7)	Medium (4.9)	High **(5.2)**	51%	NCOs
					81%	discharged 2–10 years

As shown in Table 5, the Utilitarian group scored highest on B1 (mean = 6.2). This group mainly consists of recently discharged enlisted soldiers facing financial pressures, who prioritize economic returns and make decisions based on cost-benefit calculations, thus named “Utilitarian”. Similar reasoning applies to the naming of “Mission-oriented” and “Development-focused” groups.

Table 5 further reveals systematic differences in the demographic profiles of the three groups. For example, 87% of enlisted soldiers were classified as the “Utilitarian” type, 59% of officers were categorized as the Mission-oriented type, and 51% of NCOs were grouped as the Development-focused type. Among those discharged within five years, 68% were classified as the “Utilitarian” type; among those discharged for more than ten years, 72% were categorized as the Mission-oriented type; and among those discharged between two and ten years, 81% were grouped as the Development-focused type. These characteristics collectively provide a direct and intuitive explanatory basis for understanding the differentiated motivations behind veterans’ participation in governance.

### 2.6. Policy instruments (PI) sensitivity analysis

Based on the LCA classification (Utilitarian, Mission-oriented, Development-focused) established in Section 2.5, this analysis examines the differential responses of the three groups (Utilitarian, Mission-oriented, and Development-focused) to both individual **PI** and Combined-**PI**. The evaluation encompasses four discrete policy tools (Subsidies C21, Honors C22, Training C23, Promotion C24) and three policy combinations (Subsidies+Honors C31, Training+Promotion C32, Subsidies+Training+Honors C33). This provides an empirical foundation for the subsequent development of targeted incentive strategies.

Descriptive statistics were first conducted for individual PI, with results presented in Table 6 below:

**Table 6:** Mean attractiveness scores of single PIs by group.

PI	Utilitarian (n = 269)	Mission-Oriented (n = 125)	Development-Focused (n = 131)	Full Sample (n = 525)
C21	**8.99**	7.68	7.15	8.22
C22	6.12	7.64	7.31	6.78
C23	4.31	6.18	6.01	5.18
C24	8.15	**8.07**	**8.87**	**8.31**

Table 6, through descriptive statistics, presents the mean attractiveness scores of four individual PI (C21-C24) across three veteran groups, clearly revealing their differentiated incentive preferences. The Utilitarian group rated Subsidies (C21) the highest (8.99), significantly exceeding their ratings for other instruments, reflecting a decision-making logic centered on economic returns and short-term gains. The Mission-oriented group scored notably high on Honors (C22) (7.64), while showing comparable responsiveness to Subsidies (C21) and Promotion (C24) (7.68 vs 8.07), indicating a multidimensional incentive structure with a distinct honor-driven characteristic. The Development-focused group demonstrated the strongest preference for Promotion (C24) (8.87), further validating their long-term development orientation focused on career growth. Overall, the preferences of the three groups align closely with their core group characteristics.

Descriptive analysis was subsequently conducted for Combined-PI, with comparative results shown in Table 7.

**Table 7:** Mean attractiveness scores of combined-PI.

PI Combination	Utilitarian (n = 269)	Mission-Oriented (n = 125)	Development-Focused (n = 131)	Full Sample (n = 525)
C31	9.16	8.66	8.99	9.00
C32	6.61	**9.37**	**9.47**	7.98
C33	**9.27**	8.82	9.02	**9.10**

Table 7 further illustrates the differences in attractiveness of three policy combinations (C31-C33) across different groups, revealing the synergistic effects of tool combinations and their group-specific adaptability. For the Utilitarian group, the “subsidies + recognition” combination (C31) received the highest score (9.16), indicating that adding symbolic recognition on top of material incentives yields optimal results. In contrast, the Development-focused group responded most positively to the “training + promotion” combination (C32) (9.47), demonstrating the significant advantage of this pathway in meeting both their skill enhancement and career development needs. Notably, although the comprehensive policy package (C33) scored the highest in the full sample (9.10), it provided limited additional benefit to the Utilitarian group (only a 3.1% increase compared to C31). This suggests that when designing incentive programs for this group, excessive tool stacking should be avoided, and focus should instead be placed on the most core and effective “economic-honor” combination. These results collectively indicate that the effectiveness of policy combinations is highly dependent on the specific needs of the target group, and precise matching is key to enhancing incentive efficiency.

### 2.7. Mediation analysis of behavioral expectations(BE)

Section 2.5, through Latent Class Analysis (LCA), identifies three distinct groups among veterans: Utilitarian, Mission-oriented, and Development-focused, whose BE (B1–B3) exhibit fundamental differences. Section 2.6 further reveals that these three groups also show differentiated response patterns to various PI (C21–C24) and Combined-PI (C31–C33). However, the underlying logical chain connecting group classification and policy effectiveness remains unclear—how PI influence veterans’ psychological expectations and subsequently drive their PB remains a critical question to be verified.

To address this, this section constructs and tests mediation effect models for the three groups, aiming to establish the theoretical pathway of “PI → BE→ PB” thereby validating the core hypothesis of this study: BE serve as the key psychological mediator through which PI influence PB. By selecting independent variables from the perspective of PI, mediating variables from the perspective of BE, and and dependent variables from the perspective of participation indicators for each group, and employing the Bootstrap sampling method to test the mediation effects, this section seeks to uncover the intrinsic psychological pathways through which different veteran groups respond to policies. This approach advances policy design from “differentiation based on group characteristics” to “targeted intervention based on psychological mechanisms” and provides direct empirical support for subsequent policy recommendations.

Building upon the findings from Section 2.5 and Section 2.6, we first specify the selection criteria for independent, mediating, and dependent variables in the mediation effect models for all three veteran groups, as detailed in Table 8.

**Table 8:** Variable selection for mediation models.

Utilitarian Group Model		
Variable Type	Selected Variable	Rationale
Independent Variable	Subsidies (C21)	Highest score (M = 8.99) (Table 6)
Mediator	Instrumental BE (B1)	Highest score (M = 6.2; reflects cost-benefit logic)(Table 5)
Dependent Variable	Participation frequency (D1)	Short-term economic drivers prioritize quantity over depth
**Mission-Oriented Group Model**		
Independent Variable	Honors (C22)	Exceeded material incentives (M = 7.64,) (Table 6)
Mediator	Identity-based BE (B2)	Peak score (M = 5.5); 59% officers (Table 5)
Dependent Variable	Decision-making involvement (D2)	Long-service veterans value governance voice
**Development-Focused Group Model**		
Independent Variable	Promotion (C24)	Exceptional response (M = 8.87) (Table 6)
Mediator	Developmental BE (B3)	Highest score (M = 5.2); 51% NCOs transitioning (Table 5)
Dependent Variable	Participation depth (D1 × D2)	Career-focused veterans emphasize quality

The modeling approach utilized raw scores for B1-B2-B3 and C21-C22-C23-C24, while employing the following coding scheme for D1 and D2 as specified in Table 9.

**Table 9:** Coding scheme for D1, D2, and D1 × D2.

D1	Original Options	0	1-3	4-6	>6
	Assigned Value	0	2	5	7
	Rationale	True zero point	Median value	Interval mean	Upper-bound adjustment
D2	Original Options	<20%	20-50%		>50%
	Assigned Value	1.0	1.5		2.0
	Rationale	Executive participation	Symbolic participation		Substantive participation
D1 × D2	Example: D1 = 2, D2 = 1.5,then D1 × D2 = 3				

Following the specifications in Table 8 and Table 9, mediation analyses were conducted for all three veteran groups using 500 bootstrap samples, with results presented in Table 10.

**Table 10:** Mediation analysis results across veteran groups.

Utilitarian Group (n = 269)				
Path	β	p-value	95% CI	Significance
Subsidies→Instrumental BE (A)	0.51	<0.001	[0.28, 0.74]	Significant
Instrumental BE→Frequency (B)	0.29	0.010	[0.09, 0.49]	Significant
Direct effect (c’)	0.38	<0.001	[0.22, 0.54]	Significant
Mediation effect (a × b)	**0.15**	0.008	[0.07, 0.23]	Significant
Total effect (c)=0.53		**Proportion mediated: 28.3%**		
**Mission-Oriented Group (n = 125)**				
Honors→Identity BE (A)	0.63	<0.001	[0.34, 0.92]	Significant
Identity BE→Decision-making (B)	0.41	0.002	[0.20, 0.62]	Significant
Direct effect (c’)	0.17	0.190	[-0.08, 0.42]	n.s.
Mediation effect (a × b)	**0.26**	0.002	[0.14, 0.38]	Significant
Total effect (c): 0.43		**Proportion mediated: 60.5%**		
**Development-Focused Group (n = 131)**				
Promotion→Developmental BE (A)	0.58	<0.001	[0.33, 0.83]	Significant
Developmental BE→Depth (B)	0.33	0.005	[0.13, 0.53]	Significant
Direct effect (c’)	0.09	0.130	[-0.07, 0.25]	n.s.
Mediation effect (a × b)	**0.19**	0.003	[0.09, 0.29]	Significant
Total effect (c): 0.28		**Proportion mediated: 67.9%**		

Table 10 demonstrates that the mediation effects are consistently established across all groups. For all three veteran types, both path A (PI → BE) and path B (BE → PB) reached statistical significance (p < 0.05), with all mediation effects confirmed by 95% confidence intervals (excluding zero).The effect size gradient of mediation follows: Utilitarian (0.15)<Development-focused (0.19) <Mission-oriented (0.26), while the mediation proportion gradient shows: Utilitarian (28.3%) <Mission-oriented (60.5%) <Development-focused (67.9%).

The Utilitarian group’s mechanism reveals job subsidies significantly enhance economic return expectations (β = 0.51, p < 0.001), which in turn increase participation frequency (β = 0.29, p = 0.01). The 28.3% mediation proportion reflects the “quick-fix” nature of material incentives. For Mission-oriented group, honorary recognition strongly activates identity affirmation (β = 0.63, p < 0.001), subsequently boosting decision-making involvement (β = 0.41, p = 0.002). The 60.5% mediation proportion demonstrates the purity of “honor-driven” motivation. The Development-focused group shows position promotion effectively strengthens career development expectations (β = 0.58, p < 0.001), which profoundly influence participation quality (β = 0.33, p = 0.005). The 67.9% mediation proportion underscores the centrality of career advancement needs.

### 2.8. Ethics statement

This study was conducted in accordance with standard ethical guidelines for social science research. Participation in the survey was voluntary, and all respondents provided informed consent prior to completing the questionnaire. The survey was anonymous, and no personally identifiable information was collected. As the study involved anonymous questionnaire data and posed no risk to participants, formal institutional ethical approval was not required according to local research regulations.

## 3. Discussion

### 3.1. Theoretical implications: Reconceptualizing economic rationality in veteran populations

Through the mediation analysis of three veteran archetypes, this study proposes a critical amendment to the applicability of the traditional economic rationality model within specialized populations. The findings indicate that the pure “utility-maximization” hypothesis of economic rationality holds only partially for the Utilitarian group, with a mediation proportion of 28.3%, suggesting that while their decision-making is significantly influenced by economic incentives, over seventy percent of behavioral variation is explained by non-economic factors. In contrast, the behavioral mechanisms of the Mission-oriented group (mediation proportion: 60.5%) and the Development-focused group (mediation proportion: 67.9%) are more substantially shaped by institutional values formed through military socialization and career development expectations. This result not only echoes Moskos’s(1986) institutional orientation theory [6], which posits that long-term service fosters professional ethics that transcend economic calculation, but also provides a richer “institutional-psychological” integrated perspective for understanding veteran behavior. Furthermore, the study reveals that the same policy instrument (such as position promotion) operates through differentiated psychological pathways across groups, challenging the traditional assumption of universal applicability of policy tools. Consequently, it supports the construction of a three-dimensional theoretical framework of “group characteristics-policy instruments(PI)-psychological expectations,” offering an important evidence-based supplement to public sector incentive theory derived from empirical research on a special population.

### 3.2. Policy recommendations: An “Expectation-Instrument” precision matching framework

The empirical results of this study indicate significant heterogeneity in veterans’ Behavioral Expectations (BE) and their responsiveness to Policy Instruments (PI). This finding highlights the importance of aligning incentive instruments with the psychological mechanisms underlying Participation Behavior (PB) when designing policies to promote veterans’ engagement in grassroots governance. Compared with uniform policy provision, incentive instruments should place greater emphasis on how different groups of veterans interpret and respond to specific policy signals. Based on the empirical findings derived from the Policy Instruments (PI) – Behavioral Expectations (BE) – Participation Behavior (PB) analytical framework, the following policy recommendations are proposed to enhance the effectiveness of incentive design in grassroots governance.

**Measure 1**: Propose the Establishment of a Dynamic Psychological Profiling System.The Latent Class Analysis (LCA) in this study confirms that veterans exhibit stable and classifiable heterogeneity in BE(B1-B3), clearly distinguishing three psychological archetypes: Utilitarian (51.3%), Mission-oriented (23.8%), and Development-focused (24.9%). The classification results demonstrate significant statistical discriminative power (LMR p < 0.05). However, traditional static archival management completely fails to capture such dynamic psychological characteristics, leading to a disconnect between policy provision and the actual needs of the groups. Therefore, it is essential to integrate the standardized behavioral expectation scale from this study with multidimensional practical indicators (such as family economic pressure and policy awareness) within the existing veterans’ information platform. By employing machine learning for data fusion and dynamic analysis, an automated and continuously updated individual “psychological profile” for veterans can be generated. This system can provide real-time data support for accurately identifying the three groups—Utilitarian, Mission-oriented, and Development-focused—thereby advancing the management model from “static archiving” to “dynamic individual profiling.”

**Measure 2**: Implement a Tiered and Classified Precision Incentive Scheme.Based on the three groups identified by the dynamic psychological profiling system, differentiated incentives should be designed according to the empirical results of their policy response pathways.

For the Utilitarian group, whose behavior follows the transmission logic of “Subsidies (C21) → Instrumental BE (B1) → Participation frequency (D1)” (mediation effect β = 0.15), a combined strategy of “immediate economic incentive redemption + lightweight honorary recognition” should be implemented. This includes issuing low-cost, highly visible honorary markers (C22) to validate and reinforce the optimal attractiveness of the “material-symbolic” combination (C31) for this group (score 9.16).

For the Mission-oriented group, whose behavior is dominated by the pathway “Honors (C22) → Identity-based BE (B2) → Decision-making involvement (D2)” (mediation proportion 60.5%), the core of the incentive lies in establishing an institutional mechanism for “converting honorary capital into governance authority.” This aims to transform symbolic honorary capital into substantive influence through institutionalized means.

For the Development-focused group, whose behavior heavily relies on the transmission pathway “Promotion (C24) → Developmental BE (B3) → Participation depth (D1×D2)” (mediation proportion 67.9%), it is crucial to construct an integrated pathway of “competency certification–promotion channel.” This directly addresses their highest preference for the “Training + Promotion” combination (C32) (score 9.47).

By precisely matching the incentive logic based on empirical findings with the target groups identified by the dynamic psychological profiling system, a closed loop from “precision identification” to “precision intervention” can be established. This maximizes policy effectiveness and avoids the mismatch and waste of incentive resources.

### 3.3. Research limitations

This study has three methodological limitations that require explicit acknowledgment and warrant further investigation in future research.

**The First Limitation** that in Research Design and Sample Representativeness.Although the sample covered 10 counties in Guizhou Province (n = 525), the officer subgroup accounted for only 6.7% (35 individuals), which may affect the robustness and generalizability of the findings. Additionally, the cross-sectional data could not capture the dynamic evolution of BE, making it difficult to address the important question of “whether and how individuals transition between different types.” Future research should expand the size of key subgroups through stratified sampling and employ longitudinal designs to reveal the long-term trajectories of veterans’ participation motivations.

The Second Limitation that in Measurement Tools and Model Specification.The measurement of PI attractiveness (C21-C24) may conflate “perceived importance” with “satisfaction evaluation,” leading to identification bias. Furthermore, although the mediation model verified the key mediating role of “psychological expectations,” it did not control for policy implementation quality variables (such as the qualifications of training instructors or the credibility of honorary selections). Subsequent research should develop more refined policy perception scales and incorporate implementation process indicators into the analytical framework to more comprehensively uncover the complex relationships among “policy instruments(PI), psychological mechanisms, and behavioral outcomes.”

The Third Limitation that in Exploring Policy Combinations.The study examined only three predefined Combined-PI (C31-C33) and failed to explore other potentially effective pairings (such as “Subsidies + Promotions”) or the weighting effects among different instruments (e.g., differences between a 70% Subsidies + 30% Promotions mix and an equal-weighted mix). This limits a deeper understanding of policy synergy mechanisms. It is recommended that future research employ models such as discrete choice experiments to systematically examine the interactive mechanisms of multi-instrument combinations, thereby providing more direct evidence for refined policy design.

## 4. Conclusions

By constructing the theoretical framework of “PI—BE—PB” this study systematically reveals the differentiated behavioral mechanisms of veterans’ participation in grassroots governance. Its core contribution lies in identifying and validating the existence of three veteran groups (Utilitarian, Mission-oriented, and Development-focused) and their structural differences in policy response pathways. Ultimately, this study demonstrates that the key to effectively incentivizing veterans’ participation in governance lies in building an adaptive governance system capable of identifying group psychological heterogeneity and dynamically matching PI with individual expectations. This not only addresses the practical need to enhance the effectiveness of veteran policies but also provides a transferable analytical framework for broader research on population motivation and public participation.

## Supporting information

S1 File Survey data. (XLSX)
